# First District Dental Society of New York—Clinic

**Published:** 1895-03

**Authors:** 


### PROCEEDINGS.

### First District Dental Society of New York—Clinic.

A brief report of a Clinic given by the First District Dental Society of New York, January 8, 1895. The Clinic began at 9 A. M. and continued all day. The programme announced over fifty clinicians. Owing to the great number present, it was difficult to get even a partial report of some of the operations.

Dr. J. Bond Littig, of New York City, gave a demonstration of his method of making porcelain-faced molar and bicuspid crowns. The crown was made of platinum. A portion of the labial surface was then cut out or depressed, into which he bakes porcelain with the Downie furnace, then flows onto the remaining surface Roman mat gold.

Dr. Emory A. Bryant, Washington, D. C., exhibited a removable continuous gum dental bridge, also a new clasp for plates or new removable bridges, and his methods of constructing crowns and bridge work.

Dr. Gustave P. Wiksell, Boston, Mass., exhibited Dr. Libbeys' instruments for burnishing in gold fillings. Also improved trephines, which he claims is an improvement on the Buttnier system, for shaping the ends of roots for crowns. The Doctor also demonstrated with a patient the Hayes' system of anæsthetizing sensitive dentine.

Dr. Sidney S. Stowell, Pittsfield, Mass., gave a demonstration of his rapid method of making partial gold plates, making one in eleven minutes. He takes an impression in plaster or any of the modeling compounds, into which he pours Mellotte's metal (as cool as it will flow). He then blackens the male die by burning a match underneath it to prevent the counter die from sticking to it. He then builds up the edges of the die to form a cup, into which Mellotte's metal is poured to form a counter die. Two or three thin plates, gold No. 32 gauge, are swaged separately, and then placed together and swaged again, after which the thin plates are soldered together. In this manner any thickness of plate required can be made. The doctor claims better adaptation to the mouth can be made in this manner.

Dr. S. C. G. Watkins, Montclair, N. J., is enthusiastic over his method of enlarging root-canals for their more thorough treatment, which he demonstrated. He winds cotton around a small jeweler's broach to convey the medicament, then dries the cavity and fills by again moistening it with oil of cajaput, and inserting into it a very small cone of gutta-percha and then a larger cone which has been warmed.

Dr. Z. T. Sailer, New York City, showed his dental instrument sterilizer, which is also so constructed that water is kept at an even temperature for use in the mouth, softening modeling compound, and heating a slab for softening gutta-percha stopping.

Dr. H. W. Northrup, New York City, demonstrated his method of capping teeth that have become worn down near the gum line and are sensitive. In the case presented he had inserted eighteen or nineteen crowns. There were also two or three small metal saddles which rested on the gums supported by crowns similar to the ordinary bridge.

Dr. G. A. Bowers, Nashua, N. H., demonstrated his method of making self-adjusting gold crowns, also porcelain tips for crown and bridge work.

Dr. Geo. W. Bleezarde, New York City, exhibited the Land furnace in which he baked a piece of porcelain one inch square in twenty minutes, then enameled and finised it in twenty minutes.

Dr. Joseph Speyer, of New York City, demonstrated the advantages of his soft rubber blanks for making vulcanite rubber plates. These blanks are made by pressing the soft rubber into forms that appear similar to the celluloid ones sold by dealers, and are used in the same manner, which saves the time of packing the rubber. Some of the blanks are provided with a pink rubber rim. The doctor proposes to have these blanks furnished by dealers to the profession.

Mr. A. E. Woolf, New York City, the discoverer of electrozone, was present and made some interesting experiments of the comparative action of carbolic acid and bichloride of mercury and electrozone. It was shown how, by the use of the former, albumen was coagulated, but with electrozone the albumen was completely decomposed, gas bubbles arising until the decomposition was complete. To show the non-poisonous and non-irritating effects of electrozone, Mr. Woolf drank a small portion. Electrozone is used very extensively in New York by the Board of Health for disinfecting street refuse, etc.

Dr. Lewis H. Robinson, of Brooklyn, N. Y., presented an interesting case, a girl, age fourteen, with tardy eruption of the superior incisors. The form of the teeth could be observed by pressing on the gums, but had not seemed to advance in the last twelve months.

Dr. Nelson T. Shields, New York City, described his method of making gold crowns and bridges. He is using the Downie furnace, and makes a framework of platinum and irridium bars for the bridge and the crowns of platinum to support it. These parts are then united with the smallest amount of pure gold with all excess removed with the corrundum stone. Porcelain body is then fired on to this with the Downie furnace.

Dr. V. H. Jackson, of New York City, exhibited about one hundred and thirty plaster models of special cases of irregularity of the teeth, with the appliances for their correction.

Dr. F. Messerschmitt, of New York City, demonstrated the making of Dr. V. H. Jackson's regulating appliances, showing how to carve the models, make the "Partial Clasps," and the most exact method of bending the wires, using German silver wire with soft solder, not allowing the soldering iron to remain in contact with the parts any longer than is absolutely necessary, thereby retaining all the spring of the wire.

A detailed description of constructing the appliances as given by the originator, will be found in the *Dental Cosmos* for December, 1891.

Dr. D. Genese, of Baltimore, Md., gave a demonstration of his method of attaching porcelain in cavities on the labial surfaces of the teeth.

Dr. E. Parmley Brown, of New York City, showed a "Brown

Porcelain Bridge" of twelve upper teeth, that was originally made to be worn three years, but which had been in use seven years and a half, the same having been illustrated in Dr. Evans' book on "Crown and Bridge Work."

Dr. Burton C. Russel, of Keene, N. H., gave a clinic demonstrating his method of making porcelain inlays on the labial surfaces of incisors and cuspids. He had round rods made of porcelain of varying sizes and shades. The cavity is prepared perfectly round. A rod is then chosen that is slightly larger in diameter than the cavity, and mounted in the dental engine, which is made to revolve, grinding the porcelain on a fine flat corrundum wheel in the lathe that revolves in the opposite direction. After the porcelain is perfectly fitted to the cavity, a section of it is nearly cut off, being left attached to assist in its adjustment. The inlay is then pressed into place, using oxy-phosphate of zinc. The other part is removed by breaking and the inlay ground and polished in the usual manner.

Dr. W. Irving Thayer, Williamsburgh, Mass., described his method of controlling cases of hypersensitive dentine pulpitis, acute periodontitis and chronic abscess, his method of introducing hyperdermic needle, and demonstrating the use of his medicaments Nos. 1, 2, 3 and 4.

Dr. C. Sill, of New York City, exhibited an instrument for shrinking a sub-band around a tooth for crown work. The instrument is made with a steel band that is attached outside of the metal that is to be used for the band of the crown, and by turning a screw the sub-band is made to fit the contour of the tooth.

Dr. Geo. A. Mills, of New York City, exhibited some interesting surgical and dental cases, and demonstrated Dr. Rigg's system of treatment of pyorrhœa alveolaris, and described some of the various uses of iatrol.

Dr. W. S. Twilley, Baltimore, Md., described his method of making gold and porcelain crowns similar to the Richmond system, arranged so that the porcelain fronts can be adjusted or replaced at any time. The band, cap and pin are united with solder in the usual manner. The porcelain front is then provided with a heavy backing and attached with soft wax, not bending the pins. The porcelain is then fitted to the cap and attached with hard wax, after which the porcelain is separated from the backing with a thin bladed instrument. Lead points, No. $4\frac{1}{2}$, such as are used for lead pencils made by Henry Cohen, of Philadelphia, are used in the holes caused by the withdrawal of the pins, with the ends left projecting slightly. The parts are then placed in plaster and marble dust and united with solder in the usual manner. The solder flows around the carbon points, after which the carbons are removed with a drill and the holes slightly counter-sunk. The crown is then set with oxy-phosphate of zinc and the ends of the pins riveted.

Dr. Faneuil D. Weisse, New York City, gave a brief lecture in the Amphitheatre on "Method of Diagnosing Tumors of the Buccal Cavity."

Dr. John G. Hollingsworth, Kansas City, Mo., exhibited and explained his system of crown and bridge work, and demonstrated his method of contouring gold crowns. It is reported that the system is controlled by the S. S. White Dental Manufacturing Co.

Dr. W. E. Wells, of New York City, exhibited his improved new engine mallet, and also presented his pellets for use instead of capsicum plasters.

Dr. W. C. Deane, of New York City, exhibited Power's engine mallet, made by Ash & Sons.

The Kerr furnace, manufactured by the Detroit Dental Manufacturing Co., was exhibited.

Dr. J. P. Holder, of New York City, described the use of the Edison Le Lande battery and motor for dental purposes.

